# Mechanisms of interactive specialization and emergence of functional brain circuits supporting cognitive development in children

**DOI:** 10.1038/s41539-017-0017-2

**Published:** 2018-01-10

**Authors:** Christian Battista, Tanya M. Evans, Tricia J. Ngoon, Tianwen Chen, Lang Chen, John Kochalka, Vinod Menon

**Affiliations:** 1 Department of Psychiatry & Behavioral Sciences, Stanford University School of Medicine, Stanford, CA USA; 2 Department of Neurology and Neurological Sciences, Stanford University School of Medicine, Stanford, CA USA; 3 Stanford Neuroscience Institute, Stanford University School of Medicine, Stanford, CA USA; 4 Symbolic Systems Program, Stanford University School of Medicine, Stanford, CA USA

## Abstract

Cognitive development is thought to depend on the refinement and specialization of functional circuits over time, yet little is known about how this process unfolds over the course of childhood. Here we investigated growth trajectories of functional brain circuits and tested an interactive specialization model of neurocognitive development which posits that the refinement of task-related functional networks is driven by a shared history of co-activation between cortical regions. We tested this model in a longitudinal cohort of 30 children with behavioral and task-related functional brain imaging data at multiple time points spanning childhood and adolescence, focusing on the maturation of parietal circuits associated with numerical problem solving and learning. Hierarchical linear modeling revealed selective strengthening as well as weakening of functional brain circuits. Connectivity between parietal and prefrontal cortex decreased over time, while connectivity within posterior brain regions, including intra-hemispheric and inter-hemispheric parietal connectivity, as well as parietal connectivity with ventral temporal occipital cortex regions implicated in quantity manipulation and numerical symbol recognition, increased over time. Our study provides insights into the longitudinal maturation of functional circuits in the human brain and the mechanisms by which interactive specialization shapes children’s cognitive development and learning.

## Neuroscience: brain circuits rewire with children’s cognitive development

Children’s brains rewire as their cognitive skills improve. A team led by Vinod Menon at Stanford University acquired longitudinal (i.e., multiple time points in the same individuals) functional brain imaging data while children solved arithmetic problems. Their research demonstrates that with increased experience and proficiency across development, functional brain circuits becomes more specialized. Specifically, children’s brains showed a shift from fronto-parietal circuits associated with working memory to specialized dorsal-ventral visual stream circuits that facilitate direct integration of quantity manipulation and numerical symbol recognition. Children with better problem solving skills showed increased integration of information across hemispheres in parietal cortex. This research provides new insights into how cognitive development rewires the brain and leads to specialized circuits, and contributes foundational knowledge necessary for understanding learning disabilities.

## Introduction

The human brain undergoes protracted developmental changes resulting in mature functional brain networks that engender sophisticated cognitive, problem solving, and learning abilities.^1,2^ These complex cognitive abilities in humans have been hypothesized to be supported by specialized and inter-connected functional networks that undergo protracted developmental changes in their organization between childhood and adulthood.^3^ It is now increasingly clear that understanding this requires knowledge of how dynamic interactions between distributed brain regions mature over time.^4–6^ A critical barrier to progress is the lack of task-based functional brain imaging, cognitive and behavioral data at multiple time points in the same children as they are acquiring such skills, precluding precise quantitative analysis of developmental trajectories and formation of refined functional circuits. Here, we investigate longitudinal developmental trajectories over a 6-year period spanning childhood to early adolescence, and elucidate brain mechanisms underlying specialization of functional circuits and its relation to changes in regional brain response.

The interactive specialization (IS) model provides a useful theoretical framework for investigating the formation of specialized and inter-connected functional networks over time.^1,2^ IS posits that cognitive development depends on selective strengthening of inter-regional connections over time, giving rise to specialized functional systems.^1,7,8^ More generally, functional brain maturation also involves systems-level pruning, characterized by selective strengthening of some of functional brain circuits and weakening of others.^6^ Whether IS and the formation of specialized functional circuits involves selective strengthening and weakening of functional circuits is not known.

Despite its conceptual strengths, there have been few critical tests of IS, and its instantiation at the level of functional brain circuits remains unexplored. Surprisingly, much of the formulation of the IS model to date has been based on observations of cross-sectional changes in regional brain responses rather than inter-regional interactions and plasticity of functional brain circuits.^9–12^ Although longitudinal studies are the gold standard for investigating cognitive development in children,^13–15^ the vast majority of functional brain imaging studies have been based on cross-sectional fMRI (functional magnetic resonance imaging) data. In order to make direct links to cognition and test-specific hypotheses arising from the IS model of brain development, longitudinal task-based fMRI studies are critically needed. There have only been six longitudinal developmental fMRI studies of task-related activation,^16–20^ with only one study examining changes in brain connectivity.^21^ Furthermore, extant studies of growth trajectories using longitudinal designs have focused on regional changes in brain response.^20,22^ Critically, no previous studies, either in the perceptual or the cognitive domains, have examined how specialized functional circuits emerge in children over time, and no studies have probed longitudinal changes in task-related functional circuits, which is necessary for identifying mechanisms underlying human neurocognitive development. Thus, it remains unclear how specialized functional circuits emerge from the coordination of multiple brain regions over development.

To address these gaps, we utilized a longitudinal design with multi-time point sampling to investigate the maturation of functional brain circuits supporting cognitive development and to probe the mechanisms underlying IS in the context of a numerical problem solving task. A uniquely human cognitive skill, symbolic arithmetic is especially relevant for testing the IS framework given its importance in children’s cognitive and academic skill development.^23–25^ Furthermore, this domain involves distributed brain areas whose engagement changes dynamically with skill acquisition.^26^

Core functional systems involved in symbolic arithmetic include a system for numerical quantity representation anchored in the intraparietal sulcus (IPS), and a visual number form processing system anchored in ventrotemporal occipital cortex (VTOC). Quantity-selective neurons have been found in non-human primate IPS,^27^ and fMRI adaptation paradigms have suggested that the human IPS is sensitive to quantity across stimulus formats.^28–30^ Similarly, specialization for visually presented numerals has been detected in the VTOC.^31–34^ Together, the IPS and the VTOC facilitate the efficient manipulation of numerical quantity necessary for numerical problem solving.^35^ In addition, frontal systems anchored in the insula, dorsolateral and ventrolateral prefrontal cortex (PFC) support working memory and other cognitive control functions important for effortful problem solving.^26,36^ Cross-sectional studies have suggested that IPS and VTOC activity increase with age, while PFC activity decreases with age, reflecting decreasing demands on the working memory system accompanied by increasing utilization of mature problem solving abilities.^37–39^ However, how interactions between these systems change over the course of development and which factors drive these changes are currently unknown.

**Fig. 1:**
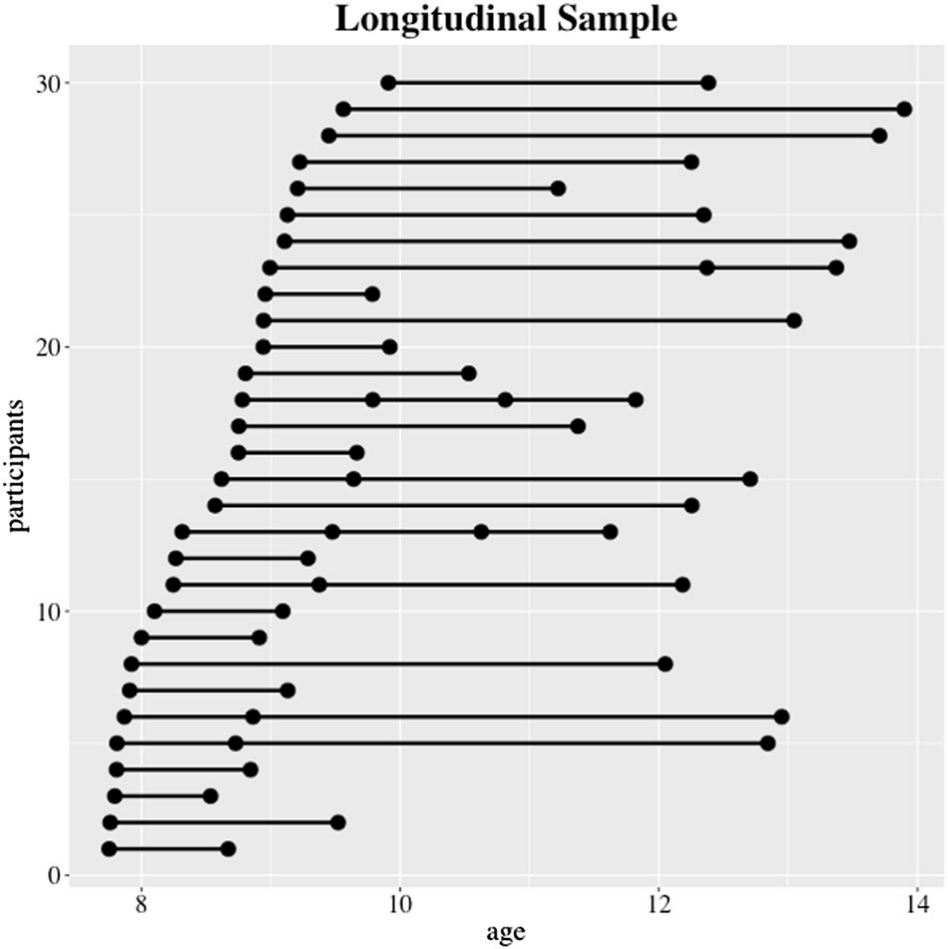
Longitudinal sampling of 30 study participants over time. Black circles represent single fMRI scan acquisitions, and multiple scans for one child are represented by interconnecting lines

We investigated developmental changes in brain connectivity in a longitudinal sample of 30 children and adolescents with task-related fMRI and cognitive data from multiple time points spanning a 6-year interval from ages 7 to 14 (Fig. 1). To better understand the mechanisms underlying IS, we examined whether longitudinal developmental changes would be associated with: (1) selective strengthening of specialized functional circuits, (2) selective weakening of PFC circuits that scaffold development, and (3) increases and decreases in activity within brain areas that show targeted changes in connectivity.^1,6^ Based on extensive evidence for its prominent role in quantity representation and manipulation,^35,40,41^ we focused on IPS connectivity to characterize longitudinal developmental changes in functional brain circuits and test-specific hypotheses about the mechanisms underlying IS. Specifically, we hypothesized that over time the IPS would show: (1) increased effective connectivity within local parietal cortical circuits; (2) increased connectivity with VTOC regions involved in basic number form processing; (3) decreased connectivity with PFC cognitive control and working memory systems. Finally, we hypothesized that this pattern of connectivity would be accompanied by selective increases in regional parietal and VTOC activity, and decreases in PFC activity, identifying a tight link between the formation of specialized large-scale brain circuits and selective changes in regional task-related activation over time.

We explicitly tested these predictions by constructing hierarchical linear models (HLMs) of developmental change in brain connectivity. HLM is ideally suited for modeling longitudinal data because it allows for the nesting of individual data, effectively accounting for individual differences.^42^ HLMs are also flexible in that they do not require that time-points be matched between children, making them a preferred choice to address the constraints and inherent difficulties of acquiring longitudinal data at multiple time points in children.^42^

## Results

### Longitudinal changes in age-normed math abilities

We first conducted an HLM analysis to characterize the profile of longitudinal changes in standardized measures of individual math abilities, assessed using the WIAT-II Numerical Operations (NumOps) subtest.^43^ By comparing a model assuming no change over age (no-change base model) and a single-factor model with age-related change in math ability (age-related change model; details in Methods), we found that the base model fitted the data better for NumOps, suggesting that age-normed measures of math abilities remained stable over time and arithmetic skill in our sample improved at a typical rate (Table 1).

**Table 1:** Hierarchical linear model (HLM) fits for longitudinal changes in behavioral measures (Fig. 2)

	ACC (%)	RT (ms)	NumOps
Fixed effects			
Intercept estimate (SE)	62.07 (13.87)	4217 (276.3)	106.0 (2.04)
Intercept *t* (*p* value)	**4.48 (9.53e-05)**	**15.26 (<2e-16)**	**52.1 (<2e-16)**
Age slope estimate (SE)	2.46 (1.06)	−179.8 (27.0)	
Age slope *t* (*p* value)	**2.31 (0.027)**	−**6.66 (2.02e-08)**	
Random effects			
Intercept variance	4683.1	76531	47.8
Slope variance	24.2		
Residual variance	50.9	1152261.1	172.1
AIC	553.56	961.0	571.7

Values for each term included in the final model and their significance are noted below. These include fixed-effect terms (regression coefficients and their standard errors) and random-effect terms (intercept variance and slope variance). Model-fitting procedures are described in the Methods section. Bold items indicate statistically significant *t* statistics. Random effects are across individuals

*SE* standard error, *AIC* Akaike information criterion, *ACC* Scanner task accuracy (% correct), *RT* Scanner task reaction time (ms), *NumOps* standard score on the numerical operations subtest of the WIAT-II

### Longitudinal changes in fMRI arithmetic task behavior

**Fig. 2:**
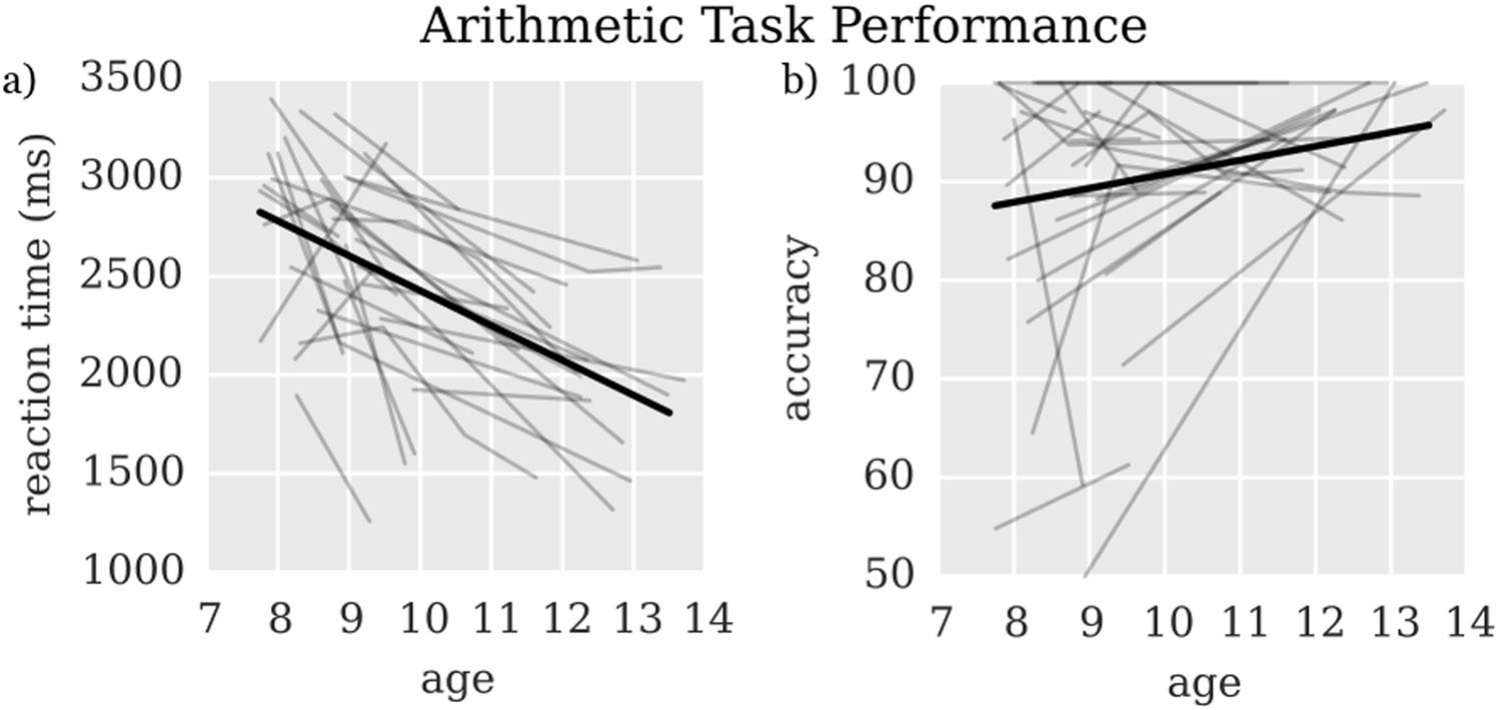
Performance improvements over time. A hierarchical linear model (HLM) was used to determine changes in **a** reaction time and **b** accuracy with age in arithmetic task performance. Dark lines show group model fits, while lighter lines show individual data from each of the 30 children

Next, we next used the same HLM analysis procedure (i.e., comparing the no-change base model and age-related change model) to characterize longitudinal changes in behavioral performance on the arithmetic task performed during fMRI acquisition. In this task, children indicated via button press whether arithmetic problems presented on the screen were correct (e.g., 2 + 4 = 6) or incorrect (e.g., 4 + 2 = 7). In contrast to the stability of NumOps, an age-normed math ability measure, accuracy on the fMRI arithmetic task increased with age at a rate of 2 %/year and reaction time decreased with age at a rate of −179.8 ms/year (Fig. 2, Table 1, model parameters in Supplementary Table 1).

### Longitudinal changes in IPS connectivity supporting arithmetic problem solving

The IPS has been shown to play a key role in quantity processing, and the development of both basic numeracy^17,44^ as well as complex numerical problem solving skills.^37^ In order to test the prediction that IPS connectivity changes over the course of development, we computed IPS functional connectivity maps derived from the arithmetic verification task relative to a number identification control task. For each participant at each age, we performed a whole-brain, voxelwise HLM analysis using procedures described in the Methods section. Briefly, we compared four types of models regarding different hypotheses on how age and math ability influence longitudinal changes in IPS connectivity: (i) the no-change base model, assuming little change in connectivity but only individual differences in connectivity strength; (ii) the single-factor model (age-related change or ability-related models), assuming that the developmental trajectories of IPS connectivity is associated with age or math ability; (iii) the two-factor model, assuming both age and math ability associated with connectivity change; and (iv) the interaction model, assuming interaction between age and ability effects. Then, based on heuristic model comparison measures, i.e., AIC (Akaike Information Criterion), we selected the best fitting model at each voxel. Our analysis focused on the left hIP3, the posterior most cytoarchitectonic subdivision of the IPS, based on its extensive role in numerical cognition^35,37–41^ as determined by meta-analysis of previously published tasks in the domain (see Methods).

**Fig. 3:**
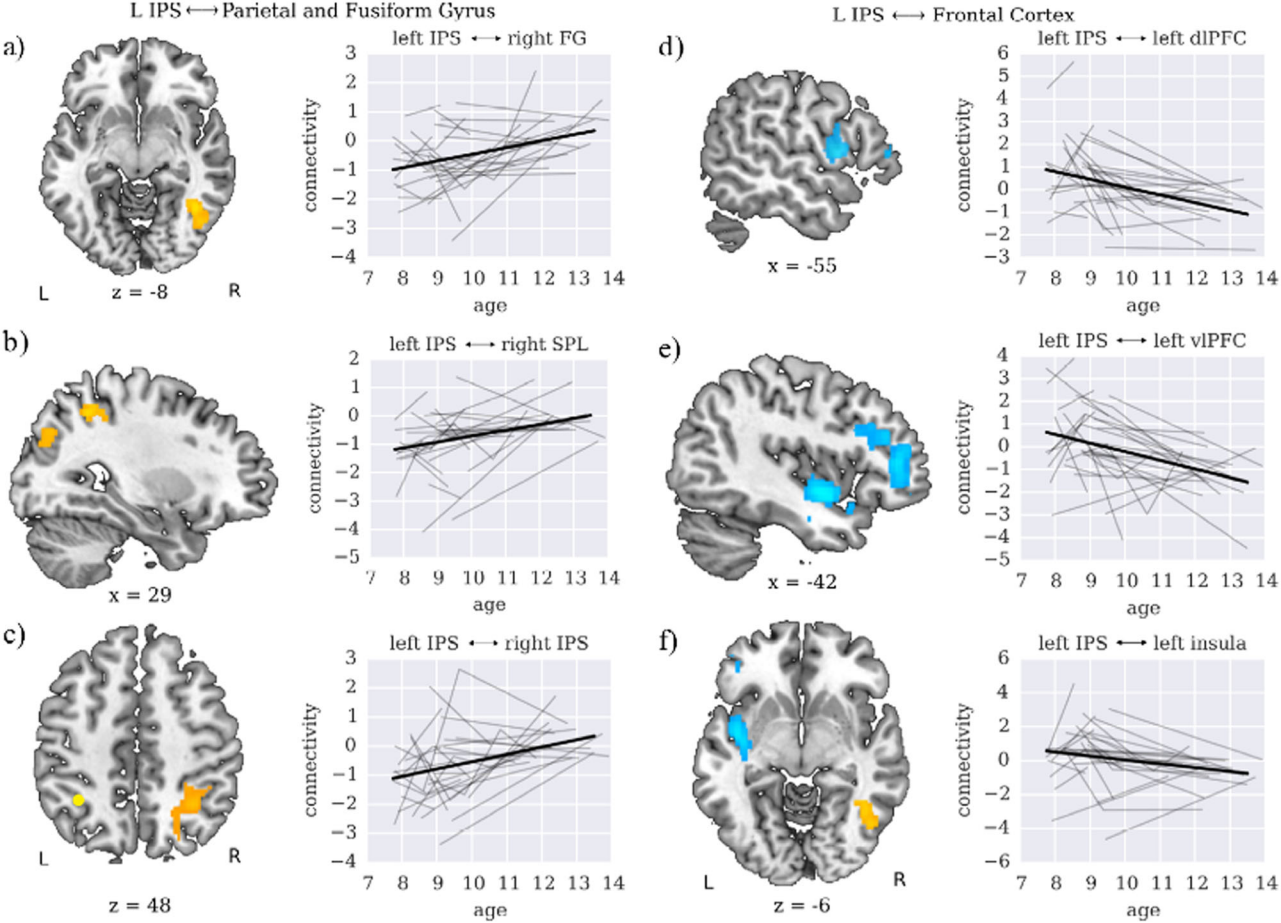
Longitudinal changes in IPS connectivity over time. Increases in **a** parietal-fusiform gyrus, **b** intra-parietal, and **c** inter-hemispheric IPS connectivity with age. Age-related decreases in **d**–**f** parietal-prefrontal cortex connectivity with age. Results of whole-brain voxelwise hierarchical linear modeling (HLM) showing brain regions with significant age-related changes in left IPS connectivity. Line plots show HLM fits for target regions extracted from the connectivity analysis. Dark lines show model fits, while lighter lines show individual participant data. IPS intraparietal sulcus, FG fusiform gyrus, SPL superior parietal lobule, dlPFC dorsolateral prefrontal cortex, vlPFC ventrolateral prefrontal cortex

This analysis revealed several clusters with significant linear age-related changes in connectivity (Fig. 3, Table 2). Left IPS connectivity with left dorsolateral prefrontal cortex (dlPFC) and left ventrolateral prefrontal cortex (vlPFC) was initially above baseline (zero) and declined with age. In contrast, left IPS connectivity with right IPS, superior parietal lobule (SPL) and fusiform gyrus (FG) were initially below baseline and increased with age.

**Table 2:** Models of longitudinal change in IPS connectivity based on whole-brain connectivity analysis (Fig. 3)

	Connectivity (gPPI)					
	L insula	L dlPFC	L vlPFC	R IPS	R SPL	R FG
Fixed effects						
Intercept Est. (SE)	2.34 (0.961)	3.55(0.797)	3.58 (0.0.971)	−3.06 (0.710)	−2.79 (0.610)	−2.77 (0.662)
Intercept t (p)	**2.43 (0.0179)**	**4.46 (4.12e-05)**	**3.61 (0.000494)**	**−4.30 (5.42e-05)**	**−4.58 (2.45e-05)**	**−4.19 (8.87e-05)**
Age slope Est. (SE)	−0.230 (0.0951)	−0.349 (0.0777)	−0.380 (0.0961)	0.252 (0.0705)	0.208 (0.0602)	0.232 (0.0656)
Age slope t (p)	**−2.41 (0.0189)**	**−4.42 (5.29e-05)**	**−0.380 (0.000211)**	**2.72 (0.00065)**	**3.46 (0.00104)**	**3.54 (0.000779)**
Random effects						
Intercept variance	0.3	0.73	0.56	0.34	0.23	0.0714
Residual variance	1.79	1.52	1.35	0.66	0.68	0.888
AIC	253.7	239.81	255.15	198.48	194.0	200.7

Values for each term included in the final model and their significance are noted below. These include fixed-effect terms (regression coefficients and their standard errors) and random-effect terms (intercept variance and slope variance). Model-fitting procedures are described in the Methods section. Bold items indicate significant *t* statistics. Random effects are across individuals

*Est.* estimate, *SE* standard error, *AIC* Akaike information criterion, *L* left hemisphere, *R* right hemisphere, *dlPFC* dorsolateral prefrontal cortex, *vlPFC* ventrolateral prefrontal cortex, *IPS* intraparietal sulcus, *SPL* superior parietal lobule, *FG* fusiform gyrus

### Longitudinal changes in IPS connectivity occur independently of changes in regional activation

In order to test the prediction that longitudinal changes in IPS connectivity are related to changes in regional activation, we analyzed longitudinal changes in regional activity among the regions that showed longitudinal changes in left IPS connectivity, comprising targets in right IPS, right SPL, right FG, right dlPFC, and left vlPFC as determined in the previous section (Fig. 3, Table 2, model parameters in Supplementary Table 2). We examined activity changes within 6 mm region of interest (ROIs) with centers at the peak voxels of each cluster obtained from the connectivity analysis. Activation within these ROIs was then averaged and used as dependent variables in HLMs (using the same model selection described above detailed in the Methods section). Age-related change in activation was not observed in any of these ROIs (Table 3, model parameters in Supplementary Table 3), suggesting that longitudinal changes in IPS connectivity occur independent of changes in regional activation. Our model fitting procedures also allowed us to test the quality of the base (intercept only) model, which indicated that activity in parietal regions was best described as consistently below baseline (significant intercept, no slope). However, PFC models did not even converge on a base model with a significant intercept. These results suggest that longitudinal changes in IPS connectivity occur independently of changes in regional activation.

**Table 3:** Models of longitudinal change in activity in target ROIs extracted from whole-brain connectivity analysis

	Regional activity					
	L insula	L dlPFC	L vlPFC	R IPS	R SPL	R FG
Fixed effects						
Intercept Est. (SE)	0.1 (0.06)	0.021 (0.0617)	0.119 (0.00673)	−0.208 (0.0531)	−0.254 (0.0512)	−0.371 (0.0685)
Intercept t (p)	1.63 (0.116)	0.34 (0.74)	1.77 (0.0814)	**−3.92 (0.000209)**	**−4.97 (2.44e-05)**	**−5.42 (9.66e-06)**
Random effects						
Intercept variance	0.0537	0.0637	8.88e-15	0.000	0.01	0.0720
Residual variance	0.158	0.113	0.312	0.19	0.16	0.154
AIC	91.7	76.1	121.5	88.9	78.3	94.3

Values for each term included in the final model and their significance are noted below. These include fixed-effect terms (regression coefficients and their standard errors) and random-effect terms (intercept variance and slope variance). Model-fitting procedures are described in the Methods section. Bold items indicate statistically significant *t* statistics. Random effects are across individuals

*SE* standard error, *AIC* Akaike information criterion, *L* left hemisphere, *R* right hemisphere, *dlPFC* dorsolateral prefrontal cortex, *vlPFC* ventrolateral prefrontal cortex, *IPS* intraparietal sulcus, *SPL* superior parietal lobule, *FG* fusiform gyrus

### Relation between parietal circuit maturation and individual differences in baseline math abilities

**Fig. 4:**
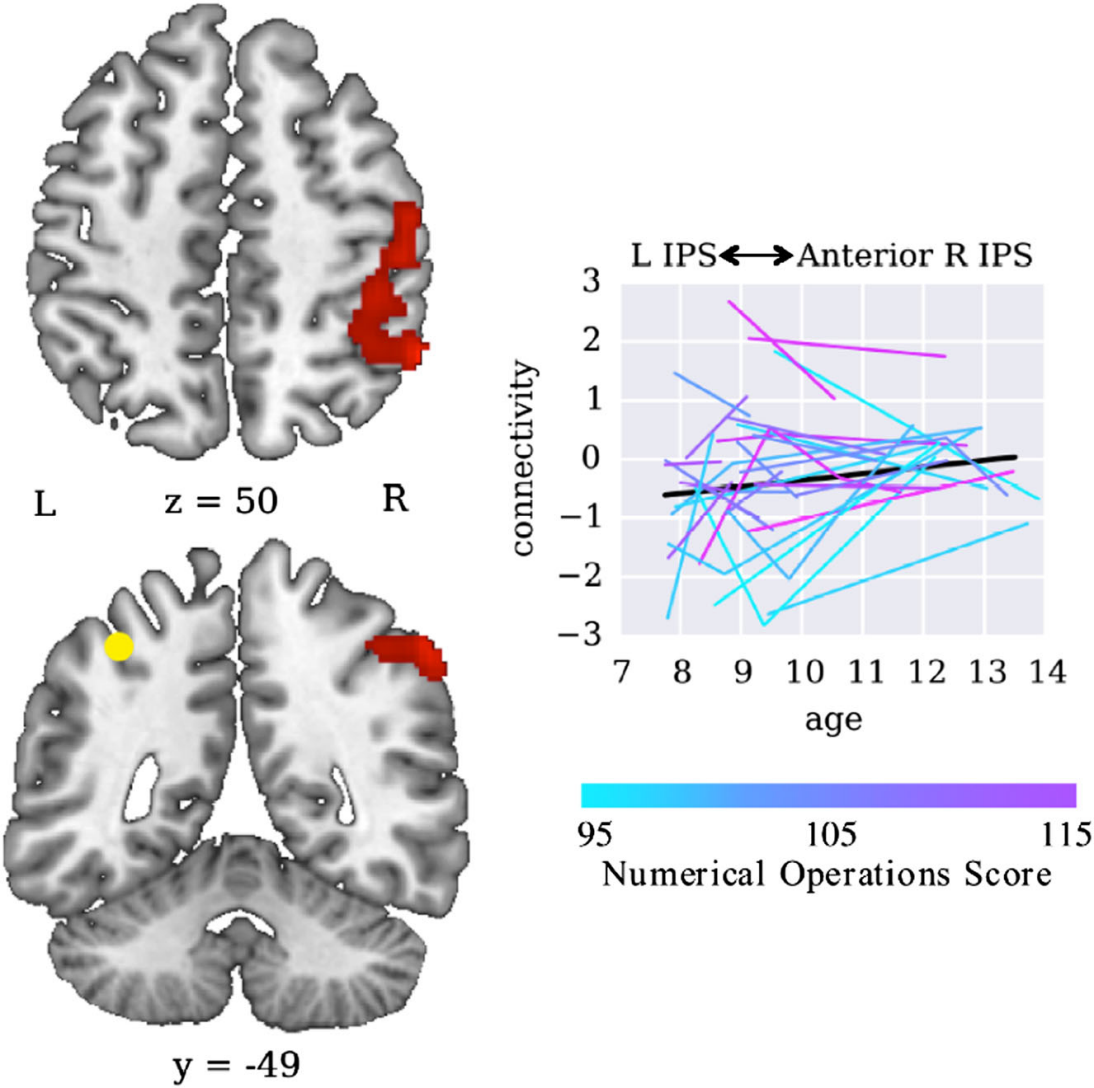
Inter-hemispheric IPS connectivity is related to individual differences in mathematical ability. Higher math ability was associated with higher inter-hemispheric IPS connectivity. Line plots show HLM fits for target regions extracted from the connectivity analysis. Colored lines show individual participant data, with light blue to purple colors indicating individual differences (low to high) in mathematical ability as measured by the NumOps subtest of the WIAT-II. Black lines show models for a hypothetical participant with NumOps score of 105 (which was roughly the mean NumOps score in the studied sample)

To examine individual differences in brain circuit maturation, we examined the relation between standardized measures of math ability and brain circuitry by computing individual intercepts using NumOps scores of the WIAT-II^43^ and left IPS connectivity. We conducted a whole-brain HLM analysis to determine whether individual NumOps scores explained additional variance in left IPS connectivity. Since NumOps scores were stable across age (Table 1), we used an average of each individual’s NumOps score across visits as a benchmark of each child’s math ability. We found one cluster in the anterior right IPS and adjoining postcentral gyrus that showed individual differences in intercept (*p* < 0.05, alpha < 0.05, cluster extent 448; Fig. 4, Table 4). This result indicates that, while all children increased in bilateral IPS connectivity across age, children with higher numerical problem solving abilities tend to maintain a consistently higher level of anterior IPS connectivity over development relative to lower achieving peers.

**Table 4:** Model of individual differences math ability and longitudinal change in L-R IPS connectivity (Fig. 4)

Connectivity (gPPI)	
	R IPS
Fixed effects	
Intercept estimate (SE)	−9.26 (3.14)
Intercept *t* (p)	**−2.95 (0.0065)**
Age slope estimate (SE)	0.11 (0.07)
Age slope t (p)	1.71 (0.092)
NumOps estimate (SE)	0.07 (0.03)
NumOps t (p)	**2.61 (0.015**)
Random effects	
Intercept variance	0.13
Residual variance	0.87
AIC	205.6

Values for each term included in the final model and their significance are noted below. These include fixed-effect terms (regression coefficients and their standard errors) and random-effect terms (intercept variance and slope variance). Model-fitting procedures are described in the Methods section. Bold items indicate significant *t* statistics. Random effects are across individuals

*SE* standard error, *AIC* Akaike information criterion, *R* right hemisphere, *IPS* intraparietal sulcus

### Specificity of brain-behavioral relationship to math skills

To examine the specificity of individual differences in brain circuit maturation to math skills, we examined the relation between standardized measures of reading ability and brain circuitry by computing individual intercepts using Word Reading scores of the WIAT-II^43^ and left IPS—right IPS connectivity. We conducted an HLM analysis to determine whether individual Word Reading scores explained additional variance in brain connectivity. We found no significant effects (Table 5), suggesting age-related increases in IPS connectivity are specifically related to math, but not reading, skills.

**Table 5:** Model of individual differences in reading ability and longitudinal change in L-R IPS connectivity

Connectivity (gPPI)	
	R IPS
Fixed effects	
Intercept estimate (SE)	−2.69 (1.75)
Intercept *t* (p)	−1.54 (0.13)
Age slope estimate (SE)	0.11 (0.07)
Age slope *t* (p)	1.60 (0.12)
Word reading estimate (SE)	0.01 (0.01)
Word reading *t* (p)	0.87 (0.39)
Random effects	
Intercept variance	0.23
Residual variance	0.86
AIC	210.2

Values for each term included in the final model and their significance are noted below. These include fixed-effect terms (regression coefficients and their standard errors) and random-effect terms (intercept variance and slope variance). Model-fitting procedures are described in the Methods section. Random effects are across individuals

*gPPI* generalized form of psychophysiological interaction, *SE* standard error, *AIC* Akaike information criterion, *R* right hemisphere, *IPS* intraparietal sulcus

### Motion and its potential impact on longitudinal changes in task-related functional connectivity

To measure head movement during fMRI scans, a rigid-body rotation and translation algorithm was implemented. Runs were excluded if any of the root mean square (RMS) *X*, *Y*, or *Z* translations exceeded 2.5 mm or if any of the RMS roll, pitch, or yaw rotations exceeded 0.1 degrees. This yielded a sample with RMS translation ranging from 0.09 mm to 2.55 mm, and RMS rotation ranging from 0.07 degrees to 3.61 degrees. HLM regressions were run to determine whether RMS translation or RMS rotation related to age, which could confound our analysis. RMS translation ranged from 0.09 mm to 2.5 mm, and RMS rotation ranged from 0.07 degrees to 3.61 degrees. Consistent with previous longitudinal developmental work,^20^ we did find age-related changes in participant head motion, with RMS translation decreasing at a rate of −0.06 mm/year, and RMS rotation decreasing at a rate of −0.09 degrees/year (model parameters in Supplementary Table 4).

An important issue in developmental studies of brain connectivity is that differences in functional connectivity levels may arise from differences in head motion, with more motion being associated to less long-range and more short-range connectivity,^45^ and these differences have confounded the results in some published research articles,^46^ creating the false impression that adults who tend to move less in the scanner than children show more long-range resting state connectivity patterns that children do. This is a concern for any connectivity analysis, but the current analysis and findings differ in two important ways. First, we used gPPI^47^ to estimate differences in task-related connectivity across conditions, in contrast to resting-state connectivity. The crucial difference being that the connectivity value for each voxel at each time point is the result of a contrast between two task conditions. Second, our findings are in a direction opposite to what has been attributed to motion. We saw decreases, rather than increases, in long-range fronto-parietal connectivity with increasing age, indicating that our connectivity results are not due to motion contamination as described in refs. ^45,46^. We reran the HLM analyses including RMS values as a covariate, and all age-related effects remain significant (Supplementary Tables 5 and 6).

## Discussion

Longitudinal investigation of functional brain circuitry provides an important window into the mechanisms underlying children’s cognitive development. Here, we probed the development of brain networks involved in problem, and implemented state of the art computational analysis via an HLM model. We examined longitudinal changes in the context of an IS model of brain development, and characterized developmental changes associated with parietal brain circuits that play a critical role in numerical problem solving. In a longitudinal cohort of children with sampling at multiple time points and ages, we found strong evidence for changes in IPS circuits characterized by both increases and decreases in task-related connectivity over time. Connectivity between left IPS and PFC decreased over time, while connectivity within posterior brain regions, including inter-hemispheric IPS connectivity and left IPS connectivity with VTOC, regions implicated in quantity manipulation and numerical symbol recognition, increased over time. Crucially, these changes occurred in the absence of changes in regional brain activation. Our findings provide insights into longitudinal growth trajectories underlying maturation of functional brain circuits and elucidate key mechanisms by which IS contributes to neurocognitive development.

### Longitudinal developmental changes in intra-parietal connectivity

Our study investigates developmental changes in parietal circuits using a longitudinal design and functional tasks designed to engage posterior parietal cortex. A notable finding here is that both intra-parietal and inter-parietal cortex functional connectivity increased over time. Specifically, both local intra-hemispheric connectivity of the left IPS with adjoining left SPL, and inter-hemispheric left-right IPS connectivity showed increases with time. Critically, inter-hemispheric IPS connectivity levels were greatest in children with the highest math ability as assessed using standardized WIAT-II measures, highlighting the behavioral significance of our findings. Although all children demonstrated increases in left-right intra-parietal connectivity, those with higher math ability maintained consistently higher levels of connectivity across time. This finding extends previous evidence relating IPS connectivity and numerical abilities in adults^48,49^ to stable inter-individual differences in inter-hemispheric IPS interactions during childhood. Our findings demonstrate that increased cross-talk of parietal circuits within and across hemispheres is a key mechanism underlying cognitive skill acquisition and the formation of specialized functional circuits during childhood.

### Longitudinal developmental changes in parietal-fusiform connectivity

We predicted that specialized processing of symbolic quantity and visual number form^34^ in the context of symbolic mental arithmetic would be reflected in increased connectivity between posterior dorsal–ventral pathways linking IPS and VTOC. Consistent with this prediction, we found that left IPS connectivity with VTOC increased over time.

This finding is noteworthy because the IPS–FG circuit is thought to be important for integrating visual number form processing in the FG with quantity processing in the IPS.^35^ Our results highlight the contribution of a previously neglected dorsal–ventral stream circuit to the development of numerical cognition, and add to the growing body of evidence for the importance of the FG and its interactions with the IPS.^50–53^ This is in line with previous work demonstrating the maturation of representations of arithmetic problems in both IPS and VTOC in adults relative to children.^54^ Our finding of IPS–FG task-related effective connectivity is also consistent with previous work demonstrating that IPS and FG structural integrity and intrinsic functional connectivity forecast growth in math skills from childhood to late childhood.^55^ The FG encompasses the visual word form area (VWFA), and recent studies suggest this VTOC subdivision is intrisically coupled with the IPS in both adults and children.^56^ It has been argued that the VWFA may not be used specifically or even predominantly for language, but rather as a general use region with processing properties that are particularly relevant to learning symbols of all types.^57^ Our findings provide evidence that this FG–IPS circuit supports the integration of visual number form processing with the parietal quantity processing system, and crucially, that effective connectivity of this circuit matures over time in parallel with problem solving abilities.

### Longitudinal developmental changes in parietal–prefrontal connectivity

We predicted that increased specialization of posterior parietal circuits would be accompanied by a decreased need for interactions with PFC. Consistent with this prediction, we found evidence for significant decreases in IPS connectivity with multiple PFC regions including the left insula, vlPFC, and dlPFC. These fronto–parietal circuits are known to play an important role in allocating resources for cognitive control and working memory during a wide range of cognitive and learning paradigms, including mental arithmetic.^35^ Our results suggest that, with increased experience, IPS regions involved in arithmetic processing rely less on interactions with working memory and cognitive control systems, as these tasks are becoming less demanding with age. Consistent with this view, greater IPS connectivity with the dlPFC, vlPFC and insula has previously been found in children with math disabilities.^58^ Notably, in contrast to the findings from the present longitudinal study spanning 6 years, a cross-sectional study of 2nd and 3rd grade children (ages 7–8 to 8–9) found no changes in IPS connectivity over the period of 1 year.^59^ Thus, the characterization of longitudinal growth trajectories over a longer time interval are necessary for detecting selective weakening of parietal-prefrontal circuits with age.

### Mechanisms of IS of functional circuits and cognitive development

The present study provides insights into the mechanisms by which IS of functional brain circuits contributes to children’s cognitive development. We summarize and synthesize key features based on the above findings. First, IS is characterized by selective strengthening of some functional brain circuits, and corresponding weakening of others. Second, IS is characterized by selective increases in local intra-hemisphere and inter-hemisphere connectivity over time in posterior brain regions known to be important for task-related information processing. In the present study, this was illustrated by increases in IPS connectivity with adjacent parietal cortex, as well as changes in inter-hemispheric IPS connectivity. Third, interactions between dorsal and ventral visual streams strengthen over time, reflecting tighter integration of different components of task-related information processing. We found evidence for increased coupling of the parietal quantity representation system with the ventral visual numerical symbol recognition system over time. Fourth, engagement of fronto–parietal circuits decreases over time during childhood, pointing to reduced need for specialized posterior brain systems to access PFC. Together, these findings provide direct evidence from a longitudinal sample for refinement of local parietal circuits, tighter integration of dorsal–ventral visual streams and reduced dependence on PFC as circuit mechanisms supporting neurocognitive development.

A strong interpretation of the IS model would predict that connectivity changes would be associated with corresponding increases or decreases in activation in interconnected brain areas. However, we found no evidence of this; none of the IPS connectivity targets in PPC, PFC or FG showed decreases, or increases in neural activity over time. Precisely how changes in functional circuits contribute to regional specialization and fine-tuned representations remains a significant challenge for future studies. It is possible that, in general, there may not be a tight link between changes in any one functional brain circuit and selective changes in regional task-related activation. An alternative hypothesis is that changes in local neuronal circuit properties (e.g., lateral inhibition) alter regional representations^60,61^ and long-range functional connectivity.^60,61^ Testing these hypotheses will require additional quantitative formalization of IS and appropriate experiments with longitudinal designs as used here.

Our findings also suggest that the overall framing of IS models needs to be refined to incorporate recent observations that multimodal association cortices, such as the IPS, subserve multiple cognitive functions. Crucially, the functions of a specific brain region depend on its context-dependent interactions with task-relevant brain regions.^35^ For example, although the numerical cognition literature has focused on the role of the IPS in quantity processing and manipulation, this same region also has a shared, flexible neural architecture for visuospatial representations and short-term memory.^61,62^ This is problematic for current framing of IS models which places an emphasis on regional specialization of domain-specific functions. We suggest that such specializations are more likely to emerge from dynamic changes in inter-regional connectivity with both increases and decreases in specific functional circuits, and their inter-relatedness, contributing to the development and maturation of cognitive skills over time.

Given the anterior to posterior shift observed in our study, and the relatively slow maturation of white matter pathways linking the PFC with parietal cortex and VTOC, we suggest that the cortical specialization observed in our study is primarily driven by learning and functional brain plasticity rather than maturation of structural connectivity. Finally, it is interesting to consider whether the type of learning and brain plasticity demonstrated here could occur on a shorter time scale. For example, would one capture similar changes in learning in a training study over hours, days or weeks? In a previous study, we found that, in typically developing children, short-term training over a course of 8 weeks on relatively simple problems, such as those used here, changes in brain activation were minimal and entirely restricted to the hippocampus.^63^ In contrast, children with dyscalculia showed marked reductions in brain responses in multiple prefrontal, parietal and VTOC regions, resulting in normalization of brain activity to levels similar to those seen in typically developing children. Thus, it appears that long-term repeated co-activation of relevant circuitry or training on more complex problems would be required to observe the patterns of normative changes observed here.

## Conclusion

In the current study we used a longitudinal design with multi-time point fMRI data and HLMs to probe the developmental trajectory of functional circuits involved in mathematical skill acquisition. Our study contributes new insights into how functional circuits linking brain systems involved in quantity manipulation, visual object recognition, and cognitive control change over time. We elucidated how dynamic reconfiguration of parietal functional circuits supports the development of mathematical skills over the course of childhood, characterized by strengthening of connections within posterior parietal cortex and between parietal cortex and ventral temporal occipital cortex, accompanied by decoupling of posterior parietal cortex from PFC. Our findings provide insights into brain mechanisms underlying IS and identify developmental shifts in connectivity levels from anterior to posterior brain systems as the strongest feature of longitudinal developmental change in individual children over time. More broadly, our study advances fundamental understanding of the circuit mechanisms by which complex cognitive abilities emerge over development.

## Methods

### Participants

We recruited children between the ages of 7 and 14 from a wide range of schools in the San Francisco Bay Area by directly mailing schools, as well as posting at libraries and elsewhere in the community. Participants were not diagnosed with any psychiatric illness, nor were they taking any medications. All protocols in the study were conducted in accordance with the American Psychological Association “Ethical Principles of Psychologists and Code of Conduct,” and approved by the Stanford University Institutional Review Board. Prior to participation, informed written consent was obtained from the legal guardian of each child, and participants independently assented to study participation. Forty-nine right-handed children who were scanned on a minimum of two time points, separated by a year, were included in the study. Fourteen participants were excluded because of excessive motion (see “Head motion and its potential impact” for details), and five participants were excluded because of MRI scanning artifacts, resulting in a final sample of *N* = 30 children with behavioral, cognitive, and functional brain imaging data for 2–4 time points per child (Fig. 1). Specifically, 23 children had two visits, five children had three visits, and two children had four visits, resulting in a total of 69 time points in the sample.

### Standardized measures of math ability

We assessed mathematical abilities with the nationally standardized achievement battery WIAT-II.^43^ It includes measures of academic skills and problem-solving abilities, and is normed by grade and academic season (i.e., summer, fall or spring). The NumOps subtest measures number writing and identification, number production, rote counting, and simple calculations (i.e., addition, subtraction, multiplication, division) via paper-and-pencil response. Example problems for 2nd and 3rd graders include: 4 − 2 = ___ (horizontal presentation) and 37 + 54 (vertical presentation).

### fMRI data acquisition

fMRI scans were run at the Stanford University Lucas Center using a custom-build head coil and a 3 T GE Signa scanner (General Electric, Milwaukee, WI). A comfortable custom-built restraint was used to minimize head movement during the scans. Using a T2* weighted gradient echo spiral in-out pulse sequence, 29 axial slices covering the whole brain (4.0 mm thickness, 0.5 mm skip) were imaged parallel to the AC–PC line.^64^ The following parameters were implemented: TR = 2 s, TE = 30 ms, flip angle = 80°, 1 interleave, FOV = 20 cm, matrix size = 64 × 64, in-plane spatial resolution = 3.125 mm. Before acquiring fMRI scans, we used an automated high-order shimming method based on spiral acquisitions to reduce blurring and signal loss from field inhomogeneity.^65^

### fMRI arithmetic task

The fMRI task had three conditions: (1) addition, (2) number identification and (3) passive fixation. During the addition condition, participants indicated, via button box, whether the answer was correct (e.g., 2 + 3 = 5) or incorrect (e.g., 2 + 3 = 6) in an addition problem with two addends. One operand ranged from 1 to 5, the other from 1 to 9, and answers were correct in 50% of the trials. Tie problems (e.g., 4 + 4 = 8) were excluded, and in incorrect trials, answers deviated by ±1 or ±2 from the correct answer.^66^ One of the addends was ‘1’ in half of the addition blocks (e.g., 5 + 1 = 6). In the number identification condition, irrelevant keyboard characters were used in place of arithmetic symbols (e.g., “2 o 3 @ 5”). Children indicated whether “5” was among the presented digits. For our analyses, we contrasted the Arithmetic condition with the control Number identification condition. This allowed us to isolate the visual and quantity processing demands that are unique to arithmetic, while removing common sensorimotor activation resulting from general task demands – a critical consideration when testing the predications of the IS framework.^1^

To optimize fMRI signal detection and the task-related functional connectivity analysis, stimuli were presented in a block design.^67^ The experimental run lasted for a total of 6 min 36 s. Stimuli were on display for 5 s, and the inter-trial interval was 500ms long. The Addition condition consisted of 36 trials, and the number identification condition consisted of 18 trials. Each of four blocks lasted either 22 or 27.5 s, consisting of either four or five trials. Block ordering was randomized across subjects, and the following constraints were in imposed: all conditions were presented in every set of four blocks, blocks of the addition condition were always separated by a block of either number identification or passive fixation, and ordering of addition and non-addition conditions were equally likely.

### fMRI preprocessing

Statistical Parametric Mapping (SPM8; http://www.fil.ion.ucl.ac.uk/spm/) was used to analyze fMRI data. To allow for T1 equilibration, the first five volumes were excluded. During reconstruction, we applied a linear shim correction for each slice separately.^64^ To correct for excessive movement, ArtRepair software was implemented.^68^ Images were realigned to correct for movement, smoothed with a 4 mm full-width half-maximum (FWHM) Gaussian kernel, and motion adjusted. Then, any deviant volumes that resulted from spikes in the global signal or sharp movement were interpolated to immediately adjacent scans, with a maximum of 10% of scan volumes being interpolated. Images were then corrected for errors in slice-timing, spatially normalized to standard MNI space, resampled to 2 mm isotropic voxels, and smoothed with a 4.5 mm FWHM Gaussian kernel.^59^ The two-step sequence of smoothing with a (1) 4-mm FWHM Gaussian kernel (implemented by the ArtRepair pipeline) and then a (2) 4.5-mm FWHM Gaussian kernel approximates a total smoothing of 6-mm FWHM Gaussian kernel (total smoothing equals the square root of the sum of the squares of individual smoothing steps).

### Individual subject analyses

#### Activation

We identified task-related brain activation by implementing the general linear model in SPM8. Volumes that were interpolated during preprocessing were flagged and de-weighted during the individual subject analyses. To account for voxel-wise latency differences in hemodynamic response, we modeled brain activity related to each task condition using boxcar functions that corresponded to the block length and convolved with a canonical hemodynamic response function and a temporal dispersion derivative. A 0.5 cycle/min high-pass filter was used to remove low-frequency drifts at each voxel. The fMRI time series was modeled as a first-degree autoregressive process to account for serial correlations. Voxel-wise effect sizes from the contrast of the Arithmetic block with the Number identification block were generated for each participant.

#### Connectivity

The “Generalized Form of Context-Dependent Psychophysiological Interactions” SPM toolbox was used to implement a generalized form of psychophysiological interaction (gPPI).^47^ gPPI allows for condition-specific estimation of task-dependent functional connectivity in multiple-condition experiments. Compared with standard PPI implementation in SPM, gPPI is more powerful, as evidenced by both simulation and empirical studies. It is also well suited for functional connectivity analysis of block design experiments,^63^ such as those featured in the current investigation.^69^ Similar to the activation step, voxel-wise effect sizes from the contrast of the Arithmetic condition with the Number identification condition were generated for each participant.

### Group analyses

#### Hierarchical linear modeling

Growth rates in both activation and connectivity were examined using HLM with the lme4 package available in R.^70^ HLM uses both multilevel fixed and random-effects analyses in order to account for within-subject nested data. It also allows for the modeling of data with varying number of time points acquired at uneven time intervals.^42^ Age was modeled as a fixed effect, while individual participants were modeled as random effects. The R package lmerTest^71^ was used for additional significance testing of models. A linear growth model with age as the growth factor and random effects for both slope and intercept is denoted for Level 1 as follows:

$${\rm outcome}_{ti} = \pi _{0i} + \pi _{1i}{\rm age}_{ti} + e_{ti}$$

$$e_{ti}\sim N\left( {0,\sigma ^2} \right)$$

and for Level 2:

$$\pi _{0i} = \beta _{00} + r_{0i}$$

$$\pi _{1i} = \beta _{10} + r_{1i}$$

In this model, *β*_00_ is the grand mean of the outcome variable at the sample’s mean age, *β*_10_ is the grand mean slope of the trajectory, and *r*_0*i*_ and *r*_1*i*_ are the random effects terms for the intercept and the slope, respectively. Estimates of each term are generated for each subject. Individual differences in outcome variables at the sample’s mean age are indicated by significant variability in *r*_0*i*_, and individual differences in slopes of outcome variables are indicated by significant differences in *r*_1*i*_.

To search for growth trends in our brain and behavioral variables, we implemented the following procedure for each outcome variable:

1. Base model. Construct a base model with a random effects term to represent subject level variance in intercept (*r*_0*i*_), but no growth. This serves as a basis for comparison with expanded models.
  $${{\rm outcome}_{ti}} = \beta _{00} + r_{0i} + e_{ti}$$
2. Single-factor models. Add a fixed effect term to represent age-related change (*β*_10_) or arithmetic ability (scores on Numerical Operations subtest), and compare the updated models to base model using *χ*^2^ test and Aikake information criterion (AIC). The significance of *β*_00_ and *β*_10_ was determined by using a *t*-test.^71^
  $${\rm outcome}_{ti} = \beta _{00} + r_{0i} + \left( {\beta _{10}} \right){\rm age}_{ti} + e_{ti}$$
  $${\rm outcome}_{ti} = \beta _{00} + r_{0i} + \left( {\beta _{10}} \right){\rm num\,ops}_{ti} + e_{ti}$$
3. Two-factor model. Expand model to include both age-related and ability-related fixed effects and compare to the best-fitting model of previous models (amongst base model and single-factor models) using *χ*^2^ and AIC.
  $${{\rm outcome}_{ti}} = \left( {\beta _{00}} + {r_{0i}} \right) + \left( {\beta _{10}} \right){\rm age}_{ti} + \left( {\beta _{20}} \right){{\rm num\,ops}_{ti}} + {e_{ti}}$$
4. Interaction model. Expand model to include the interaction between age and math ability into the model, and compare to the best-fitting model from Steps 1–3.

$$\begin{array}{*{20}{l}}{\rm outcome}_{ti} = & \left( {\beta _{00} + r_{0i}} \right) + \left( {\beta _{10}} \right){\rm age}_{ti} + \left( {\beta _{20}} \right){\rm num\,ops}_{ti}\hfill{} \\ & + \left( {\beta _{30}} \right)\ {\rm age}_{ti}^\ast {\rm num\,ops}_{ti} + e_{ti}\hfill{} \end{array}$$

When HLM was applied to whole-brain data (as in the case of connectivity analysis), the above procedure was used for each voxel, and χ^2^ tests were used to produce significance maps. Then, standard cluster-correction techniques based on Monte–Carlo simulations^72^ were used, with voxel-wise thresholds at *p* < 0.01 and a minimum cluster size of 128 voxels corresponding to a family-wise error correction for multiple comparisons (*p* < 0.01). After significant clusters were extracted, peak values from each cluster were used to create 6 mm spherical ROIs. Activation and connectivity values were extracted from these ROIs and fitted using the above procedure, and displayed in tables and figures.

### IPS seed for connectivity analysis

**Fig. 5:**
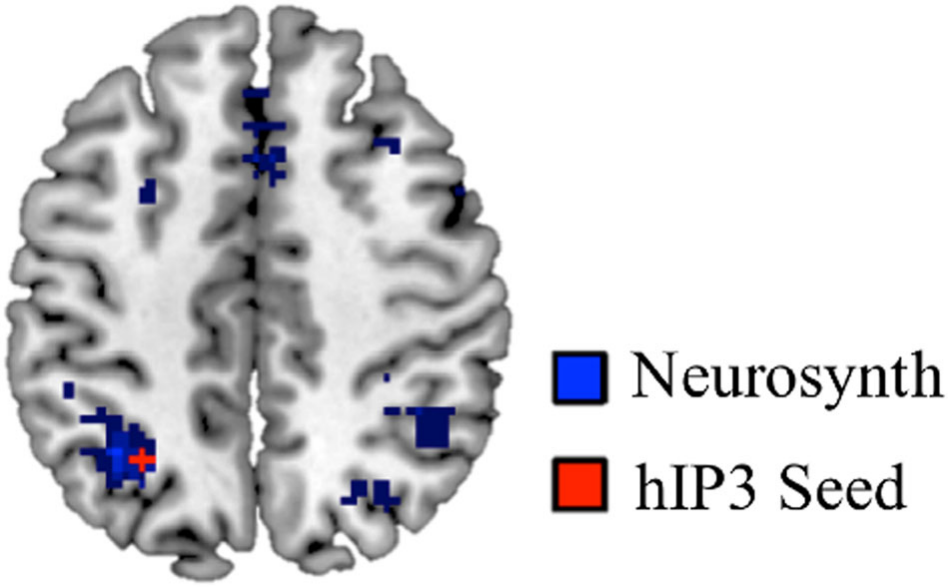
Intraparietal sulcus (IPS) region of interest (ROI). The ROI (shown in red) was derived by computing the center (*x* = −28, *y* = −58, *z* = 46) of the cytoarchitectonically-defined subdivision hIP3. This ROI shows maximal overlap with meta-analytic activation maps generated using the search term ‘arithmetic’ restricted to pediatric and adolescent subjects (shown in blue) in Neurosynth.^77^

The IPS consists of three primary cytoarchitectonically defined subdivisions, hIP1, hIP2 and hIP3,^73–75^ each with different profiles of brain activation and connectivity.^50,76^ We focused our analysis on hIP3, the most posterior subdivision, based on meta-analysis of previous task-related fMRI studies in the domain of numerical cognition. The meta-analysis was performed using Neurosynth^77^ and the search term ‘arithmetic’; to increase relevance to our longitudinal developmental study, the search was restricted to studies involving children and adolescents. The resulting activation map showed maximal overlap with cytoarchitectonically defined subdivision hIP3 of the IPS (Fig. 5).

### Data availability

The datasets analyzed during the current study are available from the corresponding author on reasonable request.

## Electronic supplementary material

Supplementary Materials

## Electronic supplementary material

**Supplementary information** accompanies the paper on the *npj Science of Learning* website (10.1038/s41539-017-0017-2).
