# Supplementary material for: Mechanisms of interactive specialization and emergence of functional brain circuits supporting cognitive development in children
Source: NPJ Sci Learn. 2018 Jan 10;3:1. doi: 10.1038/s41539-017-0017-2 (PMC6220196; doi:10.1038/s41539-017-0017-2)
Supplement: Supplementary file 1 — Supplementary Materials [file 41539_2017_17_MOESM1_ESM.pdf]

Supplementary Materials

Supplementary Table 1 - Model parameters for scanner task accuracy (ACC), Numerical Operations Standard Scores (num ops) and scanner task reaction time (RT).

Model	AIC	log likely-hood	RFX Intercept Variance	RFX Slope Variance	Residual Variance	parameter	Estimate(SE)	t(df)	p
ACC ~ 1 + (1 | PID)	594.19	-294.09	310.81		197.13	(Intercept)	87.02 (3.71)	23.44 (27.73)	< 2.00E-16
ACC ~ age + (1 | PID)	589.23	-290.62	322.37		165.06	(Intercept)	58.57 (10.97)	5.34 (53.06)	1.99E-06
						age	2.89 (1.05)	2.75 (43.86)	0.008574
ACC ~ age + (age | PID)	553.56	-270.78	4683.14	24.16	50.93	(Intercept)	62.07 (13.87)	4.48 (31.08)	9.53E-05
						age	2.46 (1.06)	2.31 (33.98)	0.02707
num ops ~ 1 + (1 | PID)	571.73	-282.86	47.80		172.10	(Intercept)	105.96 (2.04)	52.06 (29.75)	< 2.00E-16
num ops ~ age + (1 | PID)	572.58	-282.29	55.50		163.32	(Intercept)	95.75 (9.48)	10.1 (60.12)	1.42E-14
						age	1.03 (0.93)	1.1 (57.35)	0.2742
num ops ~ age + (age | PID)	572.03	-280.01	239.01	5.66	138.06	(Intercept)	90.82 (9.78)	9.28 (36.16)	4.17E-11
						age	1.57 (1.05)	1.5 (26.48)	0.1462
RT ~ 1 + (1 | PID)	987.85	-490.93	15698.45		254258.88	(Intercept)	2426.62 (67.61)	35.89 (27.76)	< 2.00E-16
RT ~ age + (1 | PID)	961.02	-476.51	76531.41		115261.11	(Intercept)	4217.09 (276.33)	15.26 (54.51)	< 2.00E-16
						age	-179.76 (27)	-6.66 (50.21)	2.02E-08
RT ~ age + (age | PID)	964.54	-476.27	2225.83	565.21	111753.67	(Intercept)	4266.68 (273.3)	15.61 (50.14)	< 2.00E-16
						age	-185.2 (27.75)	-6.67 (36.02)	8.83E-08

Supplementary Table 2 - Model parameters for connectivity ROIs.

Model	AIC	log likely-hood	RFX Intercept Var.	RFX Slope Var.	Residual	parameter	Estimate(SE)	t (df)	p
L dlPFC ~ 1 + (1 | PID)	252.64	-123.32	0.73		1.52	(Intercept)	0.15(0.22)	0.71(25.82)	0.4831
L dlPFC ~ age + (1 | PID)	238.19	-115.09	0.88		1.03	(Intercept)	3.55(0.8)	4.46(55.3)	4.12E-05
						age	-0.34(0.08)	-4.42(49.78)	5.29E-05
L dlPFC ~ age + (age | PID)	239.81	-113.91	4.64	0.02	1.01	(Intercept)	3.43(0.84)	4.07(41.86)	0.0002041
						age	-0.33(0.08)	-4.29(45.44)	9.23E-05
L insula ~ 1 + (1 | PID)	257.31	-125.66	0.31		1.95	(Intercept)	0.06(0.2)	0.33(26.04)	0.7457
L insula ~ age + (1 | PID)	253.72	-122.86	0.3		1.79	(Intercept)	2.34(0.96)	2.43(61.95)	0.01787
						age	-0.23(0.1)	-2.41(60.05)	0.01886
L insula ~ age + (age | PID)	256.02	-122.01	5.35	0.02	1.50	(Intercept)	2.34(0.98)	2.39(48.87)	0.02053
						age	-0.23(0.09)	-2.52(53.22)	0.01460
L vlPFC ~ 1 + (1 | PID)	267.23	-130.62	0.63		1.49	(Intercept)	-0.19(0.21)	-0.86(24.64)	0.3962
L vlPFC ~ age + (1 | PID)	255.15	-123.58	0.56		1.35	(Intercept)	3.57(0.97)	3.68(60.56)	0.0004941
						age	-0.38(0.1)	-3.95(58.34)	0.0002114
L vlPFC ~ age + (age | PID)	254.93	-121.46	3.44	0.28	1.21	(Intercept)	3.52(1.07)	3.29(31.11)	0.002463
						age	-0.38(0.1)	-3.78(30.91)	0.0006730
R FG ~ 1 + (1 | PID)	209.73	-101.87	0.00		1.12	(Intercept)	-0.48(0.13)	-3.73(69.0)	0.0003837
R FG ~ age + (1 | PID)	200.68	-96.34	0.07		0.88	(Intercept)	-2.77(0.66)	-4.19(62.8)	8.87E-05
						age	0.23(0.07)	3.54(61.33)	0.0007786
R FG ~ age + (age | PID)	202.14	-95.07	4.12	0.03	0.69	(Intercept)	-2.77(0.7)	-3.96(35.21)	0.00034874
						age	0.23(0.07)	3.53(40.65)	0.001060
R SPL ~ 1 + (1 | PID)	202.70	-98.35	0.17		0.86	(Intercept)	-0.73(0.14)	-5.36(27.72)	1.08E-05
R SPL ~ age + (1 | PID)	193.99	-92.99	0.23		0.68	(Intercept)	-2.79(0.61)	-4.58(59.47)	2.45E-05
R SPL ~ age + (1 | PID)						age	0.21(0.06)	3.46(56.54)	0.001037
R SPL ~ age + (age | PID)	192.49	-90.24	5.61	0.03	0.47	(Intercept)	-2.79(0.66)	-4.20(34.78)	0.0001750

R SPL ~ age + (age | PID)						age	0.21(0.06)	3.53(41.56)	0.001041
R IPS ~ 1 + (1 | PID)	204.20	-99.10	0.18		0.87	(Intercept)	-0.54(0.14)	-3.94(25.13)	0.0005692
R IPS ~ age + (1 | PID)	198.48	-95.24	0.34		0.66	(Intercept)	-2.37(0.62)	-3.83(56.71)	0.0003199
						age	0.18(0.06)	3.04(52.62)	0.003654
R IPS ~ age + (age | PID)	198.38	-93.19	4.45	0.02	0.53	(Intercept)	-2.25(0.66)	-3.41(36.64)	0.001576
						age	0.17(0.06)	2.9(43.85)	0.005835
R IPS 2 ~ age + (1 | PID)	208.91	-100.45	0.25		0.87	(Intercept)	-1.3(0.68)	-1.9(59.72)	0.06243
						age	0.11(0.07)	1.58(56.99)	0.1199
R IPS 2 ~ age + num ops + (1 | PID)	204.74	-97.37	0.13		0.87	(Intercept)	-9.26(3.14)	-2.95(26.84)	0.006477
							0.11(0.07)	1.71(60.01)	0.09248
							0.07(0.03)	2.61(24.12)	0.01535
R IPS 2 ~ age * num ops + (1 | PID)	205.64	-96.82	0.13		0.85	(Intercept)	-24.48(14.77)	-1.66(55.37)	0.1033
							1.6(1.41)	1.13(51.25)	0.2628
							0.22(0.14)	1.56(55.64)	0.1238
							-0.01(0.01)	-1.05(51.66)	0.2971

Supplementary Table 3 – Model parameters for regional brain activity in studied ROIs.

Model	AIC	log likely-hood	RFX Intercept Var.	RFX Slope Var.	Residual	parameter	Estimate(SE)	t (df)	p
L dlPFC ~ 1 + (1 | PID)	76.06	-35.03	0.06		0.12	(Intercept)	0.02(0.06)	0.34(27.25)	0.7359
L dlPFC ~ age + (1 | PID)	77.71	-34.85				(Intercept)	-0.13(0.26)	-0.49(56.75)	0.6238
						age	0.01(0.03)	0.59(52.47)	0.5558
L dlPFC ~ age + (age | PID)	75.84	-31.92	0.81	0.00	0.09	(Intercept)	-0.09(0.28)	-0.32(36.66)	0.7542
						age	0.01(0.02)	0.44(39.22)	0.6617
L insula ~ 1 + (1 | PID)	91.65	-42.83	0.05		0.16	(Intercept)	0.1(0.06)	1.63(25.52)	0.1163
L insula ~ age + (1 | PID)	93.51	-42.76				(Intercept)	0.21(0.29)	0.72(58.36)	0.4757
						age	-0.01(0.03)	-0.37(55.15)	0.7115
L insula ~ age + (age | PID)	95.42	-41.71	0.56	0.00	0.14	(Intercept)	0.24(0.3)	0.8(44.17)	0.4272
						age	-0.01(0.03)	-0.49(49.28)	0.6273
L vlPFC ~ 1 + (1 | PID)	121.52	-57.76	0.00		0.31	(Intercept)	0.12(0.07)	1.77(69)	0.08136
L vlPFC ~ age + (1 | PID)	123.39	-57.70				(Intercept)	0.25(0.38)	0.66(69)	0.5112
						age	-0.01(0.04)	-0.35(69)	0.7239
L vlPFC ~ age + (age | PID)	126.61	-57.30	0.58	0.00	0.27	(Intercept)	0.26(0.39)	0.67(44.06)	0.5044
						age	-0.01(0.04)	-0.39(49.17)	0.6978
R FG ~ 1 + (1 | PID)	94.32	-44.16	0.07		0.15	(Intercept)	-0.37(0.07)	-5.42(27.18)	9.66E-06
R FG ~ age + (1 | PID)	95.88	-43.94				(Intercept)	-0.18(0.29)	-0.61(57.45)	0.5460
						age	-0.02(0.03)	-0.67(53.57)	0.5030
R FG ~ age + (age | PID)	99.86	-43.93	0.18	0.00	0.14	(Intercept)	-0.17(0.3)	-0.55(23.01)	0.5856
						age	-0.02(0.03)	-0.71(21.49)	0.4881
R IPS ~ 1 + (1 | PID)	88.95	-41.47	0.00		0.19	(Intercept)	-0.21(0.05)	-3.92(69)	0.0002087
R IPS ~ age + (1 | PID)	90.08	-41.04				(Intercept)	0.07(0.3)	0.23(69)	0.8220
						age	-0.03(0.03)	-0.93(69)	0.3539
R IPS ~ age + (age | PID)	92.22	-40.11	0.92	0.01	0.16	(Intercept)	0.11(0.33)	0.33(27.02)	0.7476
						age	-0.03(0.03)	-0.93(24.85)	0.3593

R SPL ~ 1 + (1 | PID)	78.30	-36.15	0.01		0.16	(Intercept)	-0.25(0.05)	-4.97(30.63)	2.44E-05
R SPL ~ age + (1 | PID)	80.26	-36.13	0.01		0.16	(Intercept)	-0.2(0.28)	-0.72(65.1)	0.4743
						age	-0.01(0.03)	-0.2(64.19)	0.8442
R SPL ~ age + (age | PID)	83.06	-35.53	0.45	0.00	0.14	(Intercept)	-0.16(0.29)	-0.55(37.51)	0.5833
						age	-0.01(0.03)	-0.33(43.54)	0.7431

Supplemental Table 4 - Parameters for models of in-scanner motion.

Model	AIC	log likely-hood	RFX Intercept Var.	RFX Slope Var.	Residual	parameter	Estimate(SE)	t(df)	p
RMS_Tran ~ 1 + (1|PID)	105.37	-49.68	0.02		0.23	(Intercept)	0.64(0.06)	10(28.66)	7.40E-11
RMS_Tran ~ age + (1 | PID)	103.86	-47.93	0.03		0.21	(Intercept)	1.25(0.33)	3.82(63.23)	0.0003117
RMS_Tran ~ age + (1 | PID)						age	-0.06(0.03)	-1.9(61.75)	0.06196
RMS_Tran ~ age + (age | PID)	107.13	-47.56	0.37	0	0.2	(Intercept)	1.24(0.33)	3.72(42.34)	0.0005805
RMS_Tran ~ age + (age | PID)						age	-0.06(0.03)	-1.91(48.37)	0.06173
RMS_Rot ~ 1 + (1|PID)	125.97	-59.98	0		0.33	(Intercept)	0.76(0.07)	10.95(69)	<2.00E-16
RMS_Rot ~ age + (1 | PID)	123.0	-57.50	0		0.31	(Intercept)	1.61(0.38)	4.23(69)	7.06E-05
RMS_Rot ~ age + (1 | PID)						age	-0.09(0.04)	-2.27(69)	0.02637
RMS_Rot ~ age + (age | PID)	126.75	-57.38	0.41	0	0.29	(Intercept)	1.6(0.39)	4.1(38.89)	0.0002013
RMS_Rot ~ age + (age | PID)						age	-0.09(0.04)	-2.24(46)	0.03029
RMS_Total ~ 1 + (1|PID)	155.83	-74.92	0		0.51	(Intercept)	1.03(0.09)	11.89(69)	<2.00E-16
RMS_Total ~ age + (1 | PID)	152.69	-72.34	0		0.48	(Intercept)	2.1(0.47)	4.45(69)	3.27E-05
RMS_Total ~ age + (1 | PID)						age	-0.11(0.05)	-2.31(69)	0.02380
RMS_Total ~ age + (age | PID)	156.47	-72.24	0.58	0	0.45	(Intercept)	2.1(0.48)	4.34(39)	9.75E-05
RMS_Total ~ age + (age | PID)						age	-0.11(0.05)	-2.29(46.43)	0.02641

Supplemental Table 5 - Models of longitudinal change in IPS connectivity based on whole-brain connectivity analysis including in-scanner motion as covariate.

	Connectivity (gPPI)					
**Model**	**L insula**	**L dlPFC**	**L vlPFC**	**R IPS**	**R SPL**	**R FG**
**Fixed Effects**						
Intercept Estimate (SE)	2.03 (1.08)	2.90 (0.889)	3.20 (1.09)	-1.99 (0.701)	-2.41 (0.694)	-2.36 (0.747)
Intercept t (p)	1.87 (0.0659)	**3.259 (0.00193)**	**2.93 (0.00484)**	**-2.84 (0.00628)**	**-3.47 (0.000989)**	**-3.16 (0.00245)**
Age Slope Estimate (SE)	-0.213 (0.0981)	-0.310 (0.0796)	-0.360 (0.0990)	0.164 (0.0632)	0.188 (0.0628)	0.210 (0.0677)
Age Slope t (p)	**-2.18 (0.0336)**	**-3.899 (0.00029)**	**-3.63 (0.000596)**	**2.596 (0.0122)**	**2.99 (0.00407)**	**3.10 (0.00293)**
**Random Effects**						
Intercept variance	0.34	0.83	0.36	0.30	0.17	0.05
Residual variance	1.75	1.01	1.78	0.68	0.70	0.89
**AIC**	255.4	237.8	256.7	199.4	194.8	201.4

Supplemental Table 6 - Model of individual differences in reading ability and longitudinal change in L-R IPS connectivity including in-scanner motion as covariate.

	Connectivity (gPPI)
	**R IPS**
**Fixed Effects**	
Intercept Estimate (SE)	-8.68 (3.14)
**Intercept t (p)**	**-2.76 (0.0105)**
Age Slope Estimate (SE)	0.094 (0.068)
Age Slope t (p)	1.38 (0.172)
Num Ops Estimate (SE)	0.0724 (0.028)
**Num Ops t (p)**	**2.56 (0.0175)**
**Random Effects**	
Intercept variance	0.11
Residual variance	0.87
**AIC**	205.8

.
